# Supplementary figures and images for: The rDNA Diversity, Interseasonal Dynamic, and Functional Role of Cyanobacteria Synechococcus in the Sub-Arctic White Sea
Source: Plants (Basel). 2024 Nov 9;13(22):3153. doi: 10.3390/plants13223153 (PMC11597527; doi:10.3390/plants13223153)

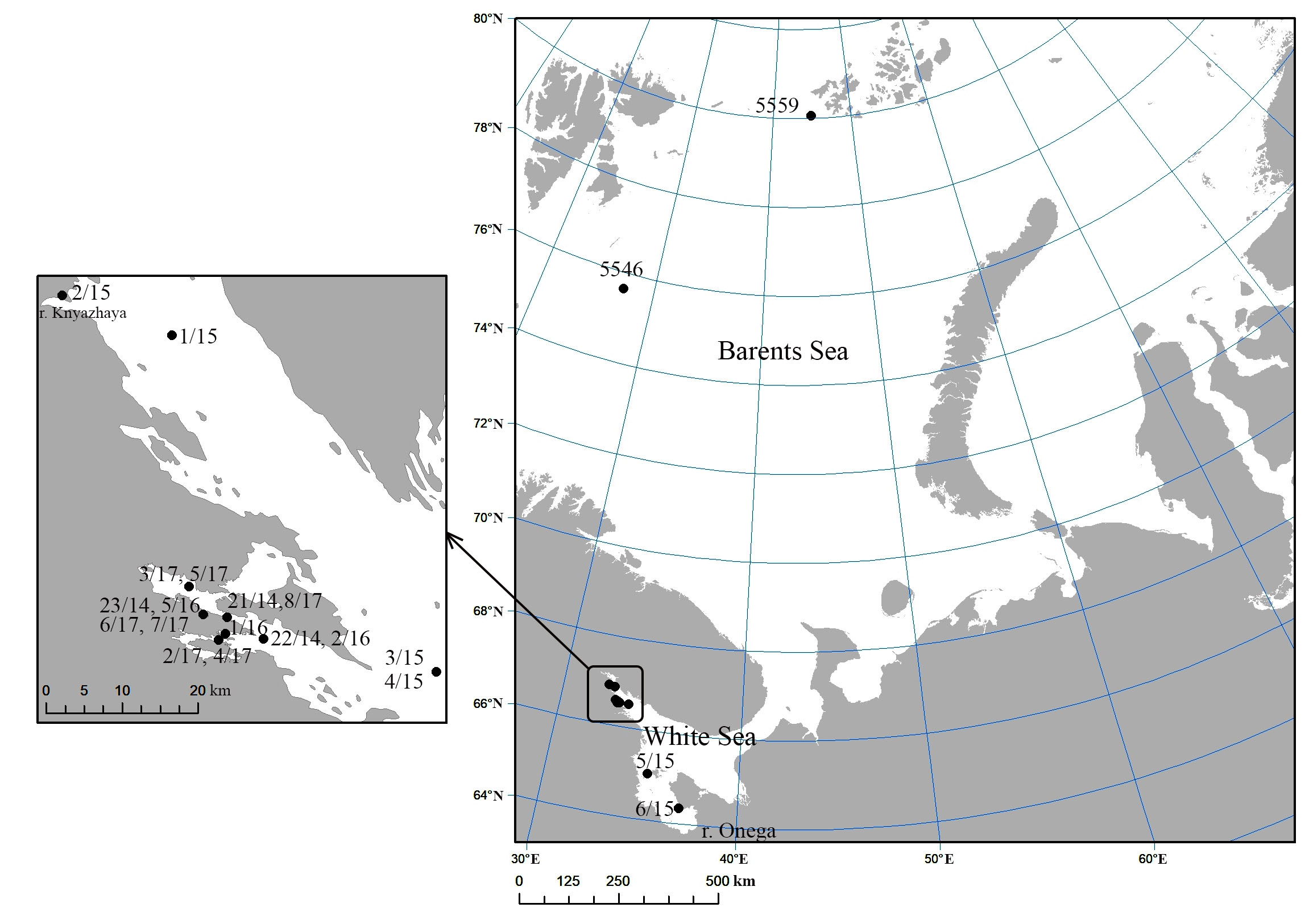

Supplement: Supplementary file 1 [file plants-13-03153-s001.zip › Figure S1.jpg]
